# Colloidal Synthesis of Sub‐1‐nm PbSe Nanowires via Cation Exchange for High‐Performance Near‐Infrared Self‐Powered Photoelectrochemical‐Type Photodetectors

**DOI:** 10.1002/advs.202501993

**Published:** 2025-04-15

**Authors:** Xiaoli Li, Sizhao Xing, Yanli Li, Guangshuo Wang, Xiaoyi Gao, Dong Li

**Affiliations:** ^1^ College of Chemical Engineering and Materials Hebei Center for New Inorganic Optoelectronic Nanomaterial Research Technology Innovation Center of Nanosized Natural Products and new materials of Hebei province Handan University Handan 056005 China; ^2^ School of Materials Science and Engineering Hebei University of Engineering Handan 056038 China; ^3^ Interdisciplinary Research Academy (IRA) Zhejiang Shuren University Hangzhou 310015 China

**Keywords:** Cation‐exchange strategy, DFT calculations, Near‐infrared photodectors, Sub‐1‐nm PbSe nanowires

## Abstract

Sub‐1‐nm nanowires (NWs) with fascinating physicochemical properties have demonstrated remarkable potential for applications across various fields. However, it remains a great challenge to prepare sub‐1‐nm NWs with near‐infrared (NIR) absorption characteristics and explore their optoelectronic applications, so far. Herein, a novel cation‐exchange strategy in N, N‐dimethylformamide (DMF) solvent is introduced to synthesize sub‐1‐nm PbSe NWs, starting from sub‐1‐nm ZnSe NWs. Theoretical calculations and nuclear magnetic resonance (NMR) measurements have confirmed that the cation exchange reaction in DMF effectively reduces the barriers for Zn^2+^ extraction and Pb^2+^ introduction compared to the conventional toluene/methanol system. Notably, the resulting sub‐1‐nm PbSe NWs exhibit a strong absorption peak at ≈940 nm. Leveraging their unique NIR absorption features and superior carrier transport properties, self‐powered photoelectrochemical‐type (PEC) photodetectors is fabricated based on sub‐1‐nm PbSe NWs embedded in polyvinylidene fluoride (PVDF) composite films. These photodetectors demonstrated exceptional photoresponse performance under 940 nm illumination (8.0 mW cm^−2^), with a typical on/off ratio of 4860, a detectivity of 4.65 × 10^11^ Jones, and a responsivity of 113 mA W^−1^. This work provides a new approach for developing and investigating NIR photoactive sub‐1‐nm semiconductor NWs with broad application prospects.

## Graphical abstract

This study presents a noval cation‐exchange approach in N, N‐dimethylformamide solvent to synthesize sub‐1‐nm PbSe nanowires, with sub‐1‐nm ZnSe nanowires as the starting material. By capitalizing on the unique near‐infrared absorption characteristics and superior carrier transport properties of these nanowires, this is successfully developed high‐performance self‐powered photoelectrochemical photodetectors based on sub‐1‐nm PbSe nanowires.

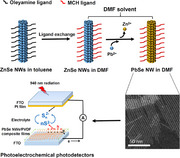

## 1 Introduction

Self‐powered photoelectrochemical‐type photodetectors (PEC PDs), a novel class of photodetection devices capable of self‐powering, have garnered significant attention in the optoelectronic field due to their low costs, straightforward manufacturing processes, and no requirement for external bias.^[^ ^1^, ^2^, ^3^, ^4^ ^]^ Recently, owing to their unique optical and electrical properties, semiconductor nanowires (NWs) have shown great promise for PEC PD and photoelectrochemical water‐splitting applications.^[^ ^5^, ^6^, ^7^, ^8^, ^9^, ^10^, ^11^, ^12^, ^13^ ^]^ Compared to their conventional nanocounterparts (typically with diameters of 1.0–5.0 nm), colloidal semiconductor NWs in the sub‐1‐nm size range have inspired great research interest owing to quantum confinement effects reaching the limit, ultrahigh structural anisotropies, ultrahigh molar extinction coefficient, and superior carrier transport property,^[^ ^14^, ^15^, ^16^ ^]^ which are regarded as the ideal building blocks to construct high‐performance PEC photodetectors and polarization‐sensitive photodetectors.^[^ ^17^, ^18^ ^]^ To date, the focus of research is primarily on most available sub‐1‐nm semiconductor NWs in the solar‐blind UV and visible regions, such as GdOOH NWs,^[^ ^17^, ^19^ ^]^ ZnS NWs,^[^ ^18^ ^]^ Bi_2_O_3_ NWs,^[^ ^20^ ^]^ CoZnCuNiFeZrCeOx NWs,^[^ ^21^ ^]^ Cu_9_S_5_ NWs,^[^ ^22^ ^]^ WO_3−x_ NWs,^[^ ^23^ ^]^ and so on. Therefore, it has been of great interest and synthetic challenge to synthesize and utilize these sub‐1‐nm semiconductor NWs with bandgaps in the infrared region.

Colloidal PbSe NWs have attracted lots of attention due to their tunable infrared bandgap in a wide range, high chemical stability, and low‐cost solution process, thus enabling them the potential as key functional materials in the infrared region.^[^ ^24^, ^25^ ^]^ Recently, a few groups reported PbSe NWs with above 4 nm diameter have been well developed by hot‐inject method,^[^ ^26^, ^27^ ^]^ but it still remains a big synthetic bottleneck to prepare sub‐1‐nm PbSe NWs. This is because there is the lack of effective means to form the PbSe nuclei below 1 nm diameter, and meanwhile to make these nuclei anisotropic growth. Among various synthetic methods, cation exchange is a convenient strategy for transforming NWs into varying composition.^[^ ^28^, ^29^, ^30^, ^31^, ^32^, ^33^ ^]^ It has been of strong interest to develop the colloidal chemistry for synthesizing sub‐1‐nm semiconductor NWs.

In this study, we present a cation‐exchange strategy in DMF solvent to prepare sub‐1‐nm PbSe NWs started with sub‐1‐nm ZnSe NWs. The solvation effects of DMF on the cation exchange reaction are elucidated and discussed through theoretical calculations and NMR measurements. Importantly, the resulting sub‐1‐nm PbSe NWs exhibit a strong absorption peak at ≈940 nm. Utilizing their unique absorption characteristics and superior carrier transport properties, we have successfully fabricated self‐powered PEC PDs based on sub‐1‐nm PbSe NWs embedded in PVDF composite films, which exhibit outstanding photodetection performance.

## 2 Results and Discussions

### 2.1 Synthesis and Characterizations of sub‐1‐nm PbSe Nanowires

**Figure 1:**
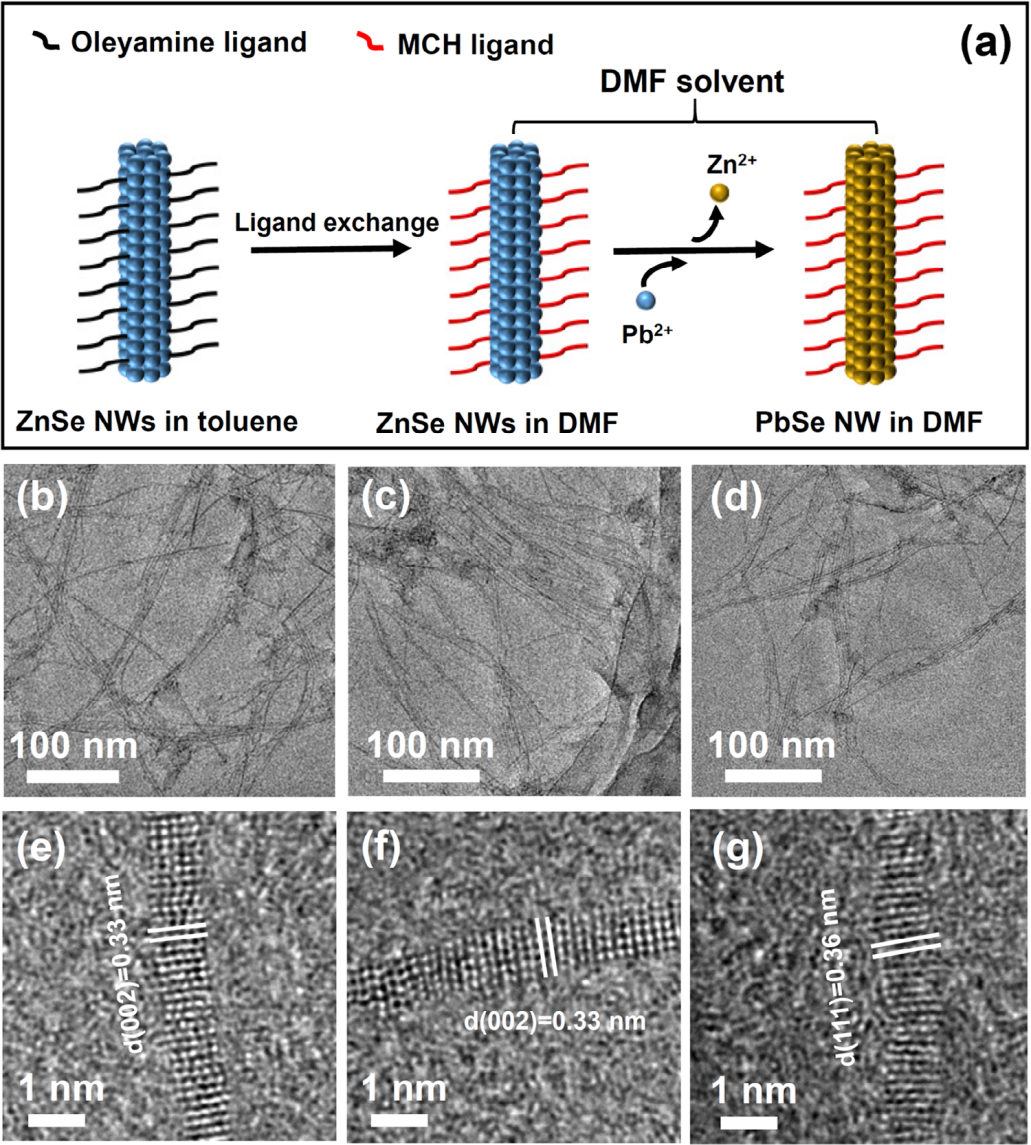
a) Schematic illustration of the cation exchange of ZnSe NWs into PbSe NWs. b) TEM images of ZnSe NWs in toluene, c) ZnSe NWs in DMF, and d) PbSe NWs in DMF after cation exchange. e) HRTEM images of ZnSe NWs in toluene, f) ZnSe NWs in DMF, and g) PbSe NWs in DMF.

**Figure** **1**a illustrates the cation‐exchange synthesis process transforming ZnSe NWs into PbSe NWs. Initially, ZnSe NWs with diameters of ≈0.9 nm (i.e., sub‐1‐nm ZnSe NWs) were synthesized following our previously reported method.^[^ ^18^ ^]^ These ZnSe NWs were subsequently transferred into the DMF phase via ligand exchange using 6‐mercaptohexanol (MCH). The MCH‐capped ZnSe NWs were then mixed with an appropriate amount of Pb^2+^ precursors (i.e., PbCl_2_) in DMF to facilitate the cation exchange reaction.

The ligand exchange and cation exchange processes of sub‐1‐nm ZnSe NWs in DMF were investigated using XPS spectra, FTIR spectra, absorption spectra, and TEM observations. XPS analyses (Figure S1, Supporting Information) consistently demonstrate that the atomic ratio of Zn to Se in the as‐synthesized sub‐1‐nm NWs is ≈1:1. This value closely matches the stoichiometric ratio in ZnSe, thereby confirming the high purity of the NWs. Figure S2a (Supporting Information) shows the FTIR spectra of ZnSe NWs before and after ligand exchange. Following ligand exchange, characteristic peaks of MCH appeared in the samples in DMF, including the OH‐stretching vibration at 3361 cm^−1^ and the CO‐stretching vibration at 1011–1052 cm^−1^. These peaks confirm the successful ligand exchange of MCH. As shown in Figure S2b (Supporting Information), the absorption spectrum of ZnSe NWs in DMF remains similar to that before ligand exchange. TEM results (Figure 1b–d) reveal that the morphology of ZnSe NWs, with diameters of ≈0.9 nm and lengths exceeding 500 nm, is well preserved during both the ligand exchange and cation exchange processes. HRTEM images (Figure 1e–g) further indicate that the original ZnSe NWs are highly crystalline and maintain their crystallinity after both ligand exchange and cation exchange. The XRD patterns (Figure S3, Supporting Information) illustrate the structural transformation from wurtzite ZnSe to cubic PbSe following the cation exchange reaction.

We further characterized the resulting sub‐1‐nm PbSe NWs using XPS, EDS, and ICP‐AES measurements. Both XPS and EDS analyses revealed that the atomic ratio of Pb to Se in the as‐prepared NWs is close to 1:1 (Figure S4, Supporting Information), which matches well with the stoichiometric ratio in PbSe and confirms their high‐purity nature. Additionally, while XPS and EDS typically provide information on surface elemental composition, ICP‐AES offers insights into the bulk elemental composition. Notably, the Pb/Se ratios obtained from XPS and EDS analyses are consistent with those determined by ICP‐AES (Table S1, Supporting Information), indicating that the ZnSe NWs were completely transformed into PbSe NWs.

### 2.2 Theoretical Calculations and NMR Measurements of the Cation Exchange Reaction

**Figure 2:**
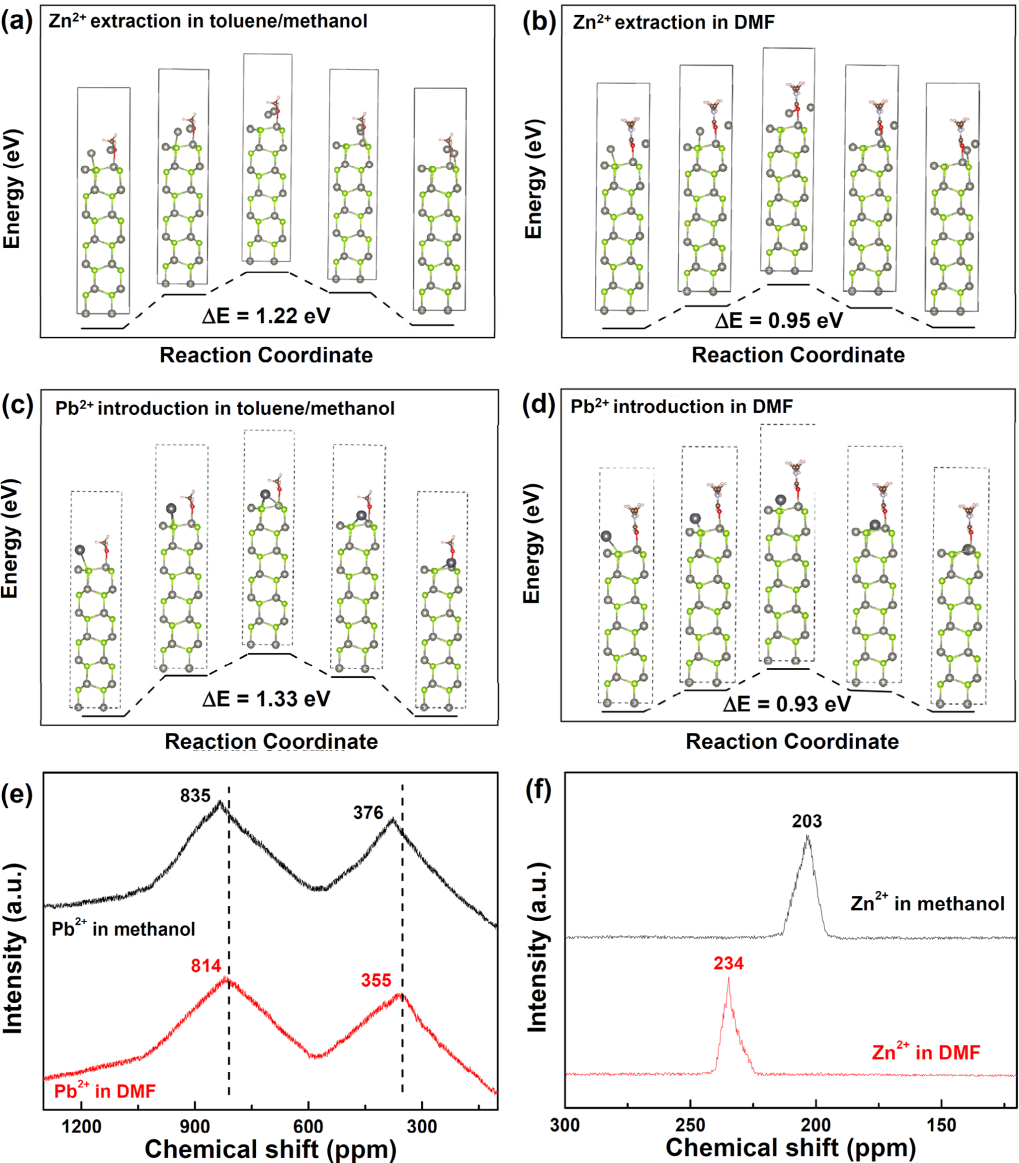
DFT calculation and NMR results of cation exchange reaction in different solvents. Activation barrier of Zn^2+^ extraction from ZnSe NWs and Pb^2+^ introduction into ZnSe NWs in toluene/methanol system a,c) and DMF solvent b,d), respectively. e) ^207^Pb and f) ^67^Zn NMR spectra of Pb^2+^ and Zn^2+^ in methanol and DMF, respectively.

To explore the solvation effects on the cation exchange of ZnSe NWs into PbSe NWs, we specifically compared the influence of DMF and toluene/methanol system on the reaction coordinates of the cation exchange reaction using density functional theory (DFT) calculation and nuclear magnetic resonance (NMR) measurements. **Figure** **2**a‐d shows the calculated activation barrier of Zn^2+^ extraction and Pb^2+^ introduction during the cation exchange reaction. The activation barrier for Zn^2+^ extraction from ZnSe NWs in the toluene/methanol system is 1.22 eV, which significantly decreases to 0.95 eV in DMF. Similarly, the activation barrier for Pb^2+^ introduction decreases from 1.33 eV in the toluene/methanol system to 0.93 eV in DMF. These results suggest that DMF solvent provides a lower activation barrier for the cation exchange reaction. To further explore the interactions between solvents and cations, we conducted NMR measurements. As displayed in Figure 2e, the chemical shifts of Pb^2+^ dissolved in DMF are 355 and 814 ppm, respectively, which shifted to 376 and 835 ppm for the methanol.^[^ ^34^ ^]^ These findings indicate that the coordination ability between DMF and Pb^2+^ is much weaker than that between methanol and Pb^2+^. Conversely, the chemical shift of Zn^2+^ dissolved in DMF is 234 ppm, which shifts to 203 ppm in methanol^[^ ^35^ ^]^ (Figure 2f). This suggests that the coordination ability between DMF and Zn^2+^ is much stronger than that between Zn^2+^ and methanol. Therefore, using DMF as the solvent reduces the activation barriers for both Zn^2+^ extraction and Pb^2+^ introduction, thereby facilitating a milder and more efficient cation exchange reaction in these NWs.

### 2.3 Optical Properties and Morphological Characterizations of PbSe NWs

**Figure 3:**
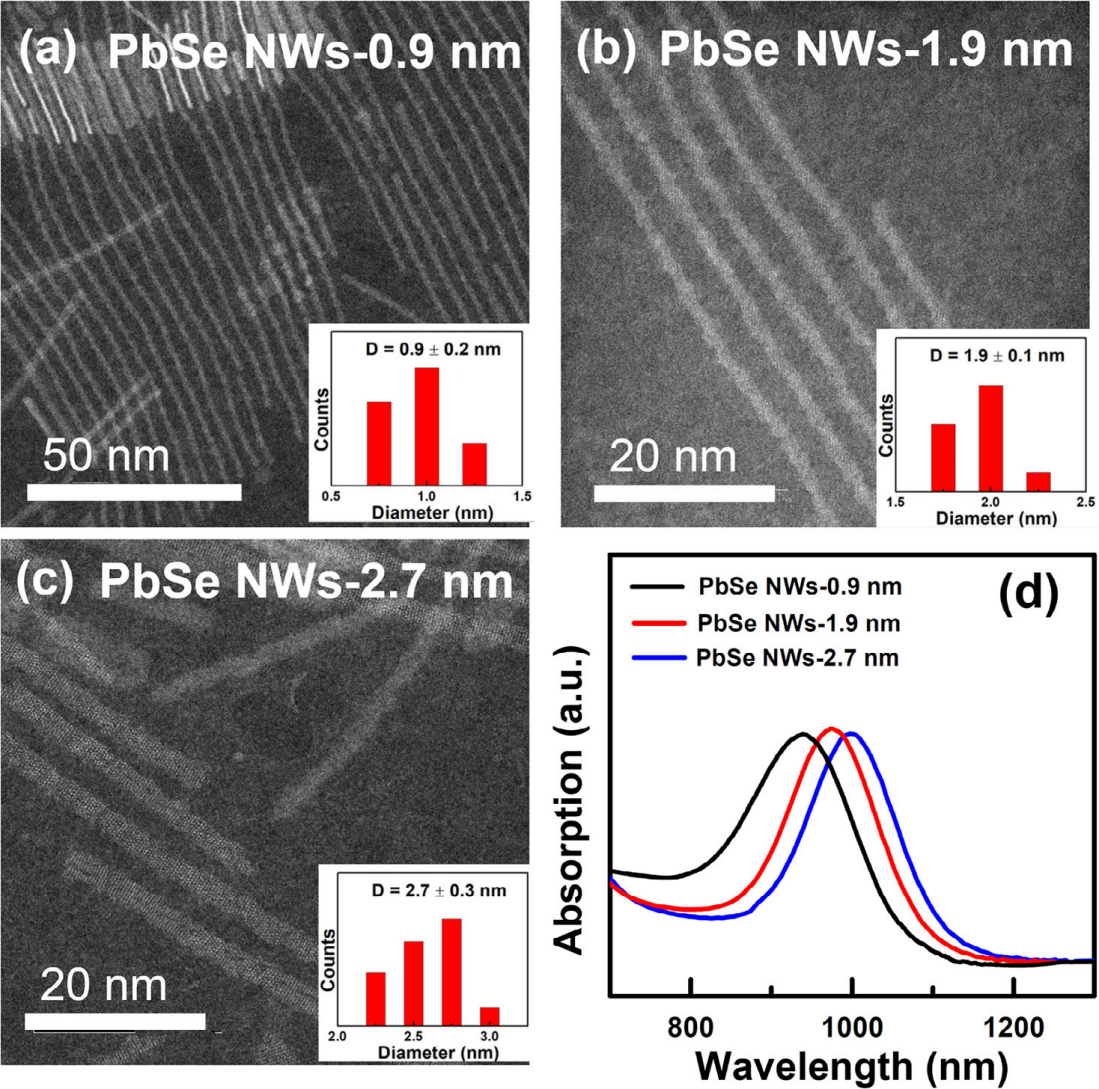
a–c) HAADF‐STEM image images of the as‐prepared PbSe NWs with average diameters of 0.9, 1.9, and 2.7 nm, respectively, and d) UV–vis‐NIR absorption spectra. The insets in (a–c) are the corresponding size distribution diagrams.

Using this synthetic approach, we successfully prepared sub‐1‐nm PbSe NWs with a diameter of 0.9 nm and an average length of 500 nm (referred to as PbSe NWs‐0.9 nm, **Figure** **3**a,d), representing the thinnest PbSe NWs reported to date. Additionally, we synthesized PbSe NWs with diameters of 1.9 nm and lengths of 500 nm (referred to as PbSe NWs‐1.9 nm, Figure 3b) and with diameters of 2.7 nm and lengths of 500 nm (referred to as PbSe NWs‐2.7 nm, Figure 3c) using the same synthetic route. Significantly, the absorption peaks of these PbSe NWs gradually redshifted from ≈940 nm (1.31 eV) to ≈1001 nm (1.23 eV) as their diameter increased from 0.9 nm to 2.7 nm (Figure 3d). This indicates that their optical absorption primarily covers the near‐infrared (NIR) region, thereby endowing them with the ability to absorb NIR light.

### 2.4 The Performance of Self‐Powered Photoelectrochemical‐Type Photodectors

**Figure 4:**
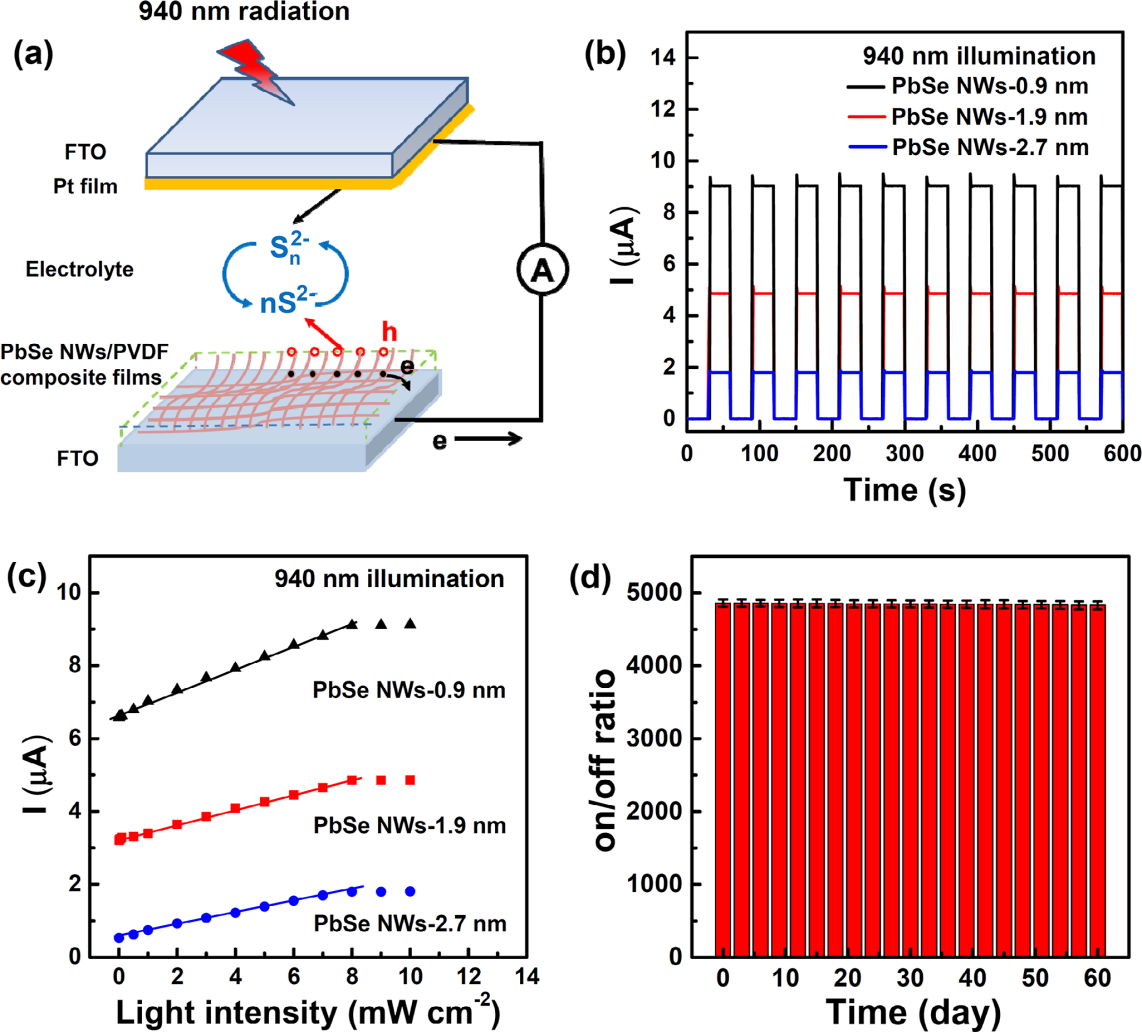
a) Schematic diagram of the device configuration of the representative self‐powered PEC PDs based on PbSe NWs. Quantitative comparison of the photoresponse performances of the PEC PDs based on the PbSe NWs with various diameters. b) Time response of the short‐circuit photocurrent of the PEC PDs based on the PbSe NWs with various diameters under 940 nm light illumination (with a power density of 8 mW cm^−2^), measured for light‐on/off states. c) Short‐circuit photocurrent density as a function of incident light intensity under 940 nm illumination. d) Storage stability of the PEC PDs based on the sub‐1‐nm PbSe NWs.

As is well known, NIR wavelengths at 940 nm are commonly used in light detection and ranging systems due to their safety for human eyes.^[^ ^36^ ^]^ Given the unique absorption properties of PbSe NWs in the NIR region, we chose PbSe NWs embedded PVDF composite films as the photoactive element to fabricate self‐powered PEC PDs with a sandwich‐type device configuration for detecting 940 nm wavelength light (**Figure** **4**a). Notably, the incorporation of the PbSe NWs in the PVDF films does not result in any shift of absorption peaks compared with solution‐dispersed counterparts (Figure S5, Supporting Information). Among the three PDs tested, the one based on PbSe NWs‐0.9 nm exhibited the best response performance under 940 nm illumination (with a power density of 8.0 mW/cm^2^). This device achieved the highest on/off ratio (≈4860), the highest photoresponsivity (≈113 mA/W, calculated using *R*_λ_ = (*I*_λ_ − *I_d_*)/*P*_λ_ *S*, where *R_λ_* is the photoresponsivity, *I_λ_* is the photocurrent, *I_d_* is the dark current, *P_λ_* is the light intensity, and *S* is the effective illuminated area), the highest detectivity (4.65 × 10^11^ Jones, calculated using D* = *R*_λ_/(2*qJ_d_*)^1/2^, where D* is the detectivity, *R_λ_* is the photoresponsivity, *q* is the elemental charge, and *J_d_* is the dark current density), the shortest rise/decay times (0.13/0.11 s), and the widest linear response range (from 2.0 µW cm^−2^ to 8.0 mW cm^−2^) (Figure 4b,c; Figures S6,S7 and S8, Supporting Information). It is worth noting that these performance metrics (i.e., on/off ratio, photoresponsivity, and detectivity) significantly outperform those of previously reported self‐powered PDs (**Table** **1**). Taking a representative PD based on PbSe NWs‐0.9 nm as an example to investigate its storage stability, we periodically measured its performance every 3 days over a period of 60 days. We found that more than 95% of the device's on/off ratio was retained after 60 days of storage (Figure 4d). This result indicates that the device possesses excellent storage stability, which can be attributed to the protective effect of the PVDF films on the NWs. Therefore, PEC PDs based on sub‐1‐nm PbSe NWs are highly promising candidates for detecting 940 nm light.

**Table 1:** Comparison of the photoresponse performances of self‐powered PEC NIR photodetectors.

Photoactive materials	Light source (nm)	Responsivity (mA W^−1^)	Detectivity (Jones)	Response time (ms)	On/off ratio	Refs.
Ag_2_Se@Ag_2_S	940	10	4.56*10^11^	700	1450	[37]
AgIn_5_Se_8_/FePSe_3_	880	3.8 × 10^−3^	5.7 *10^8^	20	350	[38]
Bi_2_O_2_S Nanosheets	850	11	8.14*10^6^	30/55	–	[39]
In_2_Se_3_ Nanosheets	850	0.13	0.11*10^9^	1/2	–	[40]
Bi_2_O_2_Se Nanosheets	850	3.7 × 10^−2^	0.13*10^8^	100/140	–	[41]
Te/Se nanocomposites	980	–	3.3 × 10^8^	80/180	–	[42]
TeSe nanorods	808	–	7.6 × 10^5^	–	–	[43]
Sub‐1‐nm PbSe NWs	940	113	4.65 × 10^11^	130/110	4860	This work

### 2.5 The Absorption Capacities and Carrier Transport Properties of PbSe NWs

**Figure 5:**
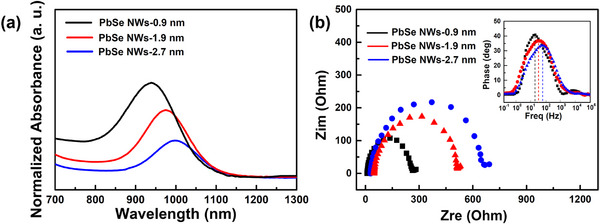
a) Normalized absorption spectra of PbSe NWs with different diameters. b) Nyquist plots of the PDs based on PbSe NWs with different diameters under 940 nm illumination. The inset in b is the corresponding Bode plot.

In order to clarify why the PDs based on the PbSe NWs‐0.9 nm could achieve excellent photoresponse performance, we explore the absorption capacities and carrier transport properties of the PbSe NWs of various diameters (e.g., 0.9, 1.9, and 2.7 nm). As shown in **Figure** **5**a, the normalized absorbance of the PbSe NWs‐0.9 nm is higher than that of the other two thicker NW samples in the NIR region. This enhanced absorption is likely due to the significant quantum confinement effect in the PbSe NWs‐0.9 nm, which results in a higher oscillator strength concentrated at the excitonic peak compared to the thicker NW counterparts. This, in turn, endows them with superior NIR absorption ability. We further compared their carrier transport properties using EIS studies. As displayed in Figure 5b, EIS spectra indicate that, under 940 nm illumination, the PD based on the PbSe NWs‐0.9 nm exhibits the smallest charge transfer resistance (ca. 255 Ω) and the longest electron lifetime (5.6 ms, calculated using, where *f_max_* is the characteristic frequency of charge transfer at NWs/electrolyte interface^[^ ^44^, ^45^ ^]^) among the three PDs. This demonstrates that the PbSe NWs‐0.9 nm possess the best carrier transport characteristics. Therefore, we conclude that the combination of excellent absorption ability and superior carrier transport properties of the sub‐1‐nm PbSe NWs enables the PDs based on them to achieve such remarkable photoresponse performance.

## 3 Conclusion 

In summary, we have developed a cation‐exchange synthesis method for sub‐1‐nm PbSe NWs using sub‐1‐nm ZnSe NWs as a starting template in DMF solvent. The choice of DMF solvent significantly reduces the activation barriers for Zn^2+^ extraction and Pb^2+^ introduction during the cation exchange reaction, as demonstrated by theoretical calculations and NMR measurements. Notably, the resulting sub‐1‐nm PbSe NWs exhibit a strong absorption peak at ≈940 nm. Leveraging their exceptional NIR absorption properties and superior carrier transport characteristics, we successfully fabricated self‐powered PEC PDs based on sub‐1‐nm PbSe NWs embedded in PVDF composite films. Under 940 nm illumination (8.0 mW cm^−2^), these photodetectors exhibited remarkable photoresponse characteristics, with a typical on/off ratio of 4860, a detectivity of 4.65 × 10^11^ Jones, and a responsivity of 113 mA W^−1^. To the best of our knowledge, this is the first report on the facile synthesis of high‐quality sub‐1‐nm PbSe NWs and the development of high‐performance NIR PDs based on this material. Our findings pave the way for the controllable preparation and optoelectronic device applications of this novel nanomaterial.

## 4 Experimental section

### Chemicals

Zinc nitrate hexahydrate (Zn(NO_3_)_2_·6H_2_O), methanol, N,N‐dimethylformamide (DMF), and toluene were purchased from Beijing Chemical Reagent Company. Lead chloride (PbCl_2_), Sulfur (S) powder, Selenium (Se) powder (200 mesh, 99.99%), sodium sulfide nonahydrate (Na_2_S·9H_2_O, 99.9%), sulfur powder, sodium hydroxide (NaOH), potassium chloride (KCl), H_2_PtCl_6_, polyvinylidene fluoride (PVDF, Mn = 150 000), oleylamine (OLA) were obtained from Aladdin Chemistry Co., Ltd. 6‐mercaptohexanol (MCH) were obtained from Alfar Aesar. All reagents were of analytical grade and used without further purification.

### Synthesis of ZnSe NWs

Sub‐1‐nm ZnSe NWs (with a diameter of 0.9 nm) were synthesized following Li's previous report.^[^ ^18^ ^]^ In detail, 0.2–0.5 mmol Zn(NO_3_)_2_ and 0.2−0.5 mmol Se powder were separately predissolved in 10 mL and 5.0 mL of OLA in a glovebox. Subsequently, the two precursor solutions were combined and transferred to a Teflon‐lined stainless‐steel autoclave (25 mL) at room temperature, and then heated at 120 °C for 2.0−12 h. Post‐reaction, the prepared ZnSe samples were purified via a conventional washing, precipitation, and redispersion process, and ultimately dissolved in toluene or dried under vacuum for further characterization. Likewise, ZnSe NWs with diameters of 1.9 and 2.7 nm were produced using the same procedure, with the reaction temperature increased to 140 and 160 °C, respectively.

### Ligand Exchange of ZnSe NWs

Typically, 100 mg of purified OLA‐capped sub‐1‐nm ZnSe NWs were added into 15 mL of DMF and degassed with N_2_ three times at room temperature for preparing MCH‐capped ZnSe NWs. Then, 1 mL of MCH was quickly injected into the system. The mixture was allowed to stand for 30 min, during which it gradually became transparent from its initial turbidity. The MCH‐capped ZnSe NWs were then washed several times with toluene and finally dissolved in DMF or dried under a vacuum for further use and characterization. Similarly, the ligand exchange reaction of ZnSe NWs with diameters of 1.9 and 2.7 nm were carried out using the same procedure.

### Cation Exchange Synthesis of PbSe NWs in DMF

To synthesize sub‐1‐nm PbSe NWs, 20 mg purified sub‐1‐nm ZnSe NWs capped with MCH were dissolved into 15 mL of DMF with 50 mg of PbCl_2_. The solutions were stirred at 60 °C under a N_2_ atmosphere. After 40 mins at 60 °C, the reaction was terminated. The PbSe NWs were washed several times with toluene and then dissolved in DMF or dried under vacuum for further characterization. Similarly, PbSe NWs with diameters of 1.9 and 2.7 nm were prepared using the same procedure.

### Fabrication of Self‐Powered PEC PDs Based on PbSe NWs

To fabricate PbSe NW film‐based photoanodes, 200 mg of PbSe NWs and 0.4 g of PVDF was uniformly dispersed in 6 mL of DMF solution after sonication for 15 min. Then, the prepared PbSe NWs with different diameters were then embedded into PVDF composite films and spin‐coated onto fluorine‐doped tin oxide (FTO) glass. They were subsequently treated at 70 °C for 24 h in a vacuum oven. Platinized counter electrodes were prepared by spin‐coating a 4.5 mM isopropanol solution of H_2_PtCl_6_ onto FTO glass, followed by heating at 450 °C for 15 min. The PbSe NW film‐based photoanodes and the platinized counter electrodes were finally assembled into a sandwich‐type device configuration. Their interelectrode spaces were filled with a liquid electrolyte composed of 0.5 m Na_2_S, 2.0 m S, 0.2 m KCl, and 0.5 m NaOH in methanol/water (7:3 by volume).

### Normalization of the Amounts of the PbSe NWs Dispersed in DMF

To compare the absorbances and extinction coefficients of PbSe NWs with different diameters in DMF, the amounts of PbSe NWs need to be normalized accordingly. Specifically, this was first extracted 1 mL of DMF solution containing purified PbSe NWs and then digested them into a standard aqueous solution. Subsequently, the Pb^2+^ concentration in the standard solution (i.e., the total Pb atom number in the purified nanowire product or Total Pb Amount) was obtained by ICP measurement. Meanwhile, the Pb atom number in each nanowire (Pb Atom Number per NW) can be determined based on the information of Pb atom number in each unit cell, the size of the unit cell (the lattice constant *a* = 6.126 Å), and the size of the PbSe NW by TEM observation. Therefore, the amount of PbSe NWs (NW Amount) with various diameters and similar lengths in the testing solution can be determined by the following equation:

(1) NWAmount=TotalPbAmount/PbAtomNumberperNW

### Theoretical Calculations

All calculations were carried out for the material in the framework of Density Functional Theory (DFT) using the Vienna Ab initio Simulation Package (VASP 6.3.0).^[^ ^46^, ^47^ ^]^ The generalized gradient approximation (GGA) of the Perdew‐Burke‐Ernzerhof (PBE) function was used to describe the exchange‐correlation energy.^[^ ^48^ ^]^ The projected augmented wave (PAW) method and pseudopotentials were used to describe the interactions between valence electrons and ions.^[^ ^49^ ^]^ To ensure the efficiency of the computational results and parallel computing. A 2*2*1 k‐point grid under Monkhorst‐Pack was used in the optimisation process and 450 eV truncation energy is set. The lattice parameters and ionic positions of all crystals were fully relaxed, and the convergence criteria for the total energy of all relaxed atoms and the final force were 10^−5^ eV and 0.05 eV Å^−1^, respectively. In addition, CL‐NEB method was used to study the dynamics of lithium ion migration. This method mainly uses linear interpolation, inserting five intermediate states between the reactants and products to find the minimum activation energy of the reaction.

### Characterizations

UV–vis‐NIR absorption spectra were recorded using a UV‐3600 spectrophotometer. Fourier transform infrared (FTIR) spectra were obtained on a PE2000 FTIR with a resolution of 4 cm^−1^. Transmission electron microscopy (TEM), high‐angle annular dark‐field scanning transmission electron microscopy (HAADF‐STEM), and high‐resolution TEM (HRTEM) observations were performed using a FEI G20 transmission electron microscope operated at 200 kV, equipped with an energy disperse X‐ray spectrometer (EDS). ^207^Pb and ^67^Zn nuclear magnetic resonance (NMR) experiments were conducted on a Bruker Ascend 700 spectrometer, using a 5 mm quartz tube and deuterated solvents of DMF‐d7. All of the ^207^Pb and ^67^Zn NMR chemical shifts were referenced to tetramethyllead and Zn(NO_3_)_2_, respectively. X‐ray diffraction (XRD) patterns were recorded using a Rigaku D/max‐2400 diffractometer operated at 40 kV and 200 mA current under Cu K*a* radiation (l = 1.5418 Å). XPS data were obtained on an ESCALAB MK spectrometer equipped with a hemisphere analyzer. The samples for XRD and XPS measurements were all purified solid powders. The photocurrent responses of PbSe NWs‐based photodetectors were tested using a controlled intensity modulated photoresponse spectroscopy system (CIMPS, Zahner, PP211) coupled with a Zahner Zennium electrochemical workstation, with intensity‐modulated light emitting diodes (940 nm) as light sources. Electrochemical impendence spectroscopy (EIS) was performed on a Zahner Zennium electrochemical workstation with a sweeping frequency from 100 mHz to 1 MHz at open‐circuit voltage with 10 mV AC amplitude.

## Conflict of Interest

The authors declare no conflict of interest.

## Supporting information



Supporting Information

## Data Availability

The data that support the findings of this study are available from the corresponding author upon reasonable request.
