# Supplementary material for: Colloidal Synthesis of Sub‐1‐nm PbSe Nanowires via Cation Exchange for High‐Performance Near‐Infrared Self‐Powered Photoelectrochemical‐Type Photodetectors
Source: Adv Sci (Weinh). 2025 Apr 15;12(26):2501993. doi: 10.1002/advs.202501993 (PMC12245030; doi:10.1002/advs.202501993)
Supplement: Supplementary file 1 — Supporting Information [file ADVS-12-2501993-s001.docx]

Supporting Information

Colloidal Synthesis of Sub-1-nm PbSe Nanowires via Cation Exchange for High-Performance Near-infrared Self-powered Photoelectrochemical-type Photodetectors

Xiaoli Li, Sizhao Xing, Yanli Li, Guangshuo Wang*, Xiaoyi Gao* and Dong Li*


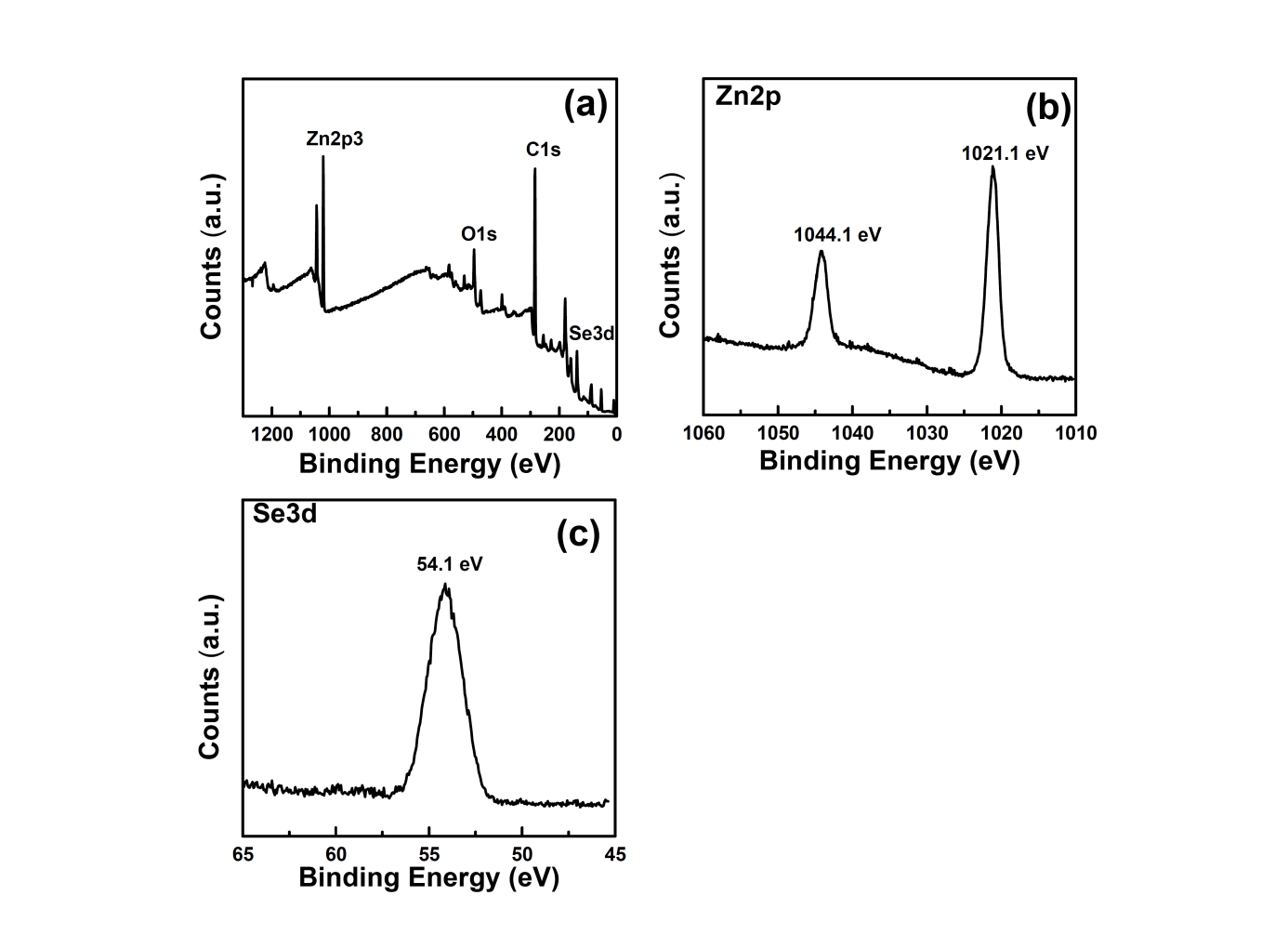


**Figure S1.** Full-range XPS spectrum (a), XPS spectrum of Zn_2p_ core level (b), and Se_3d_ core level (c) of resulting ZnSe NWs. The peak at 1044.1 eV, 1021.1 eV and 54.1 eV correspond to the binding energies of Zn2p 3/2, Zn2p1/2 and Se3d core level, respectively. Quantitative calculation of (b) and (c) gives the atomic ratio of Zn to Se in the NWs is close to 1:1, which is in good agreement with the stoichiometric ratio in ZnSe.^[1]^


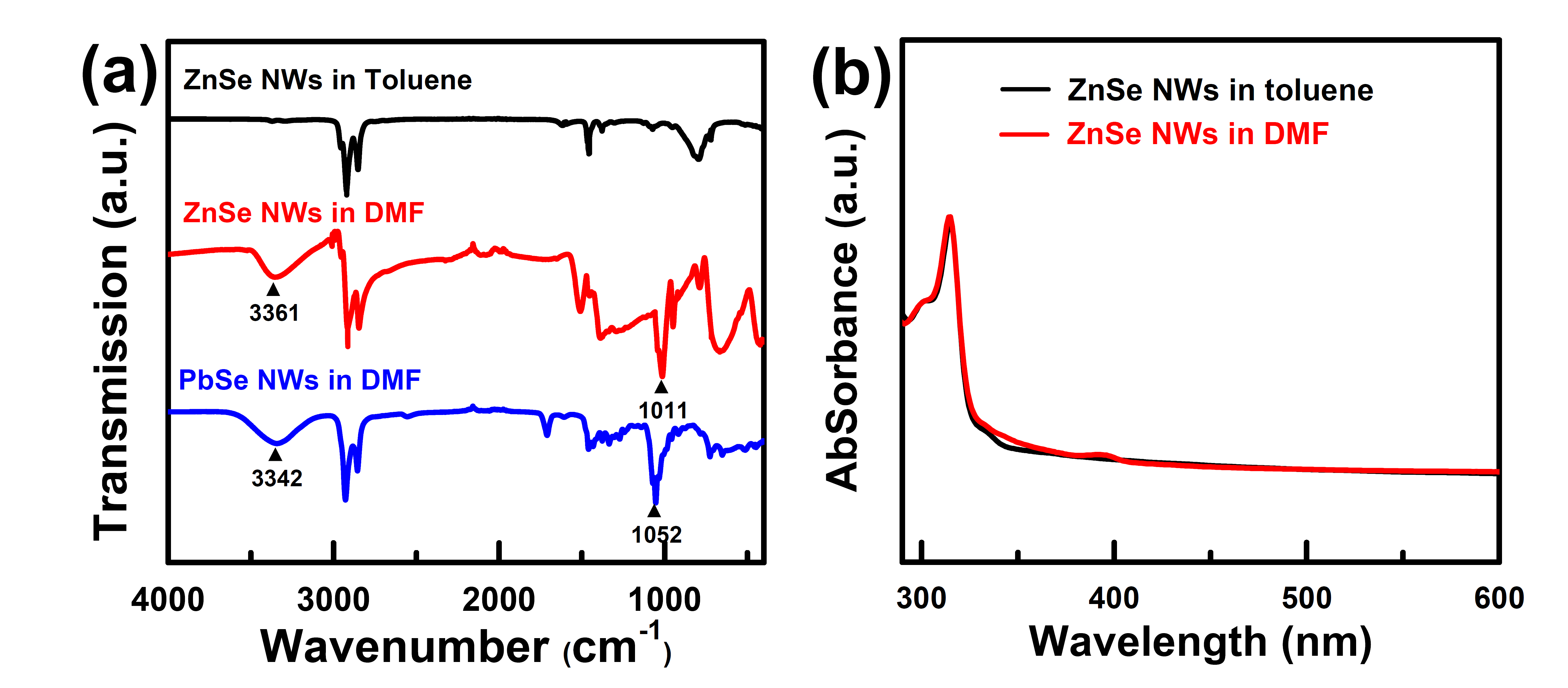


**Figure S2.** FTIR spectrum (a) and absorption spectra (b) of ZnSe NWs before and after ligand exchange.


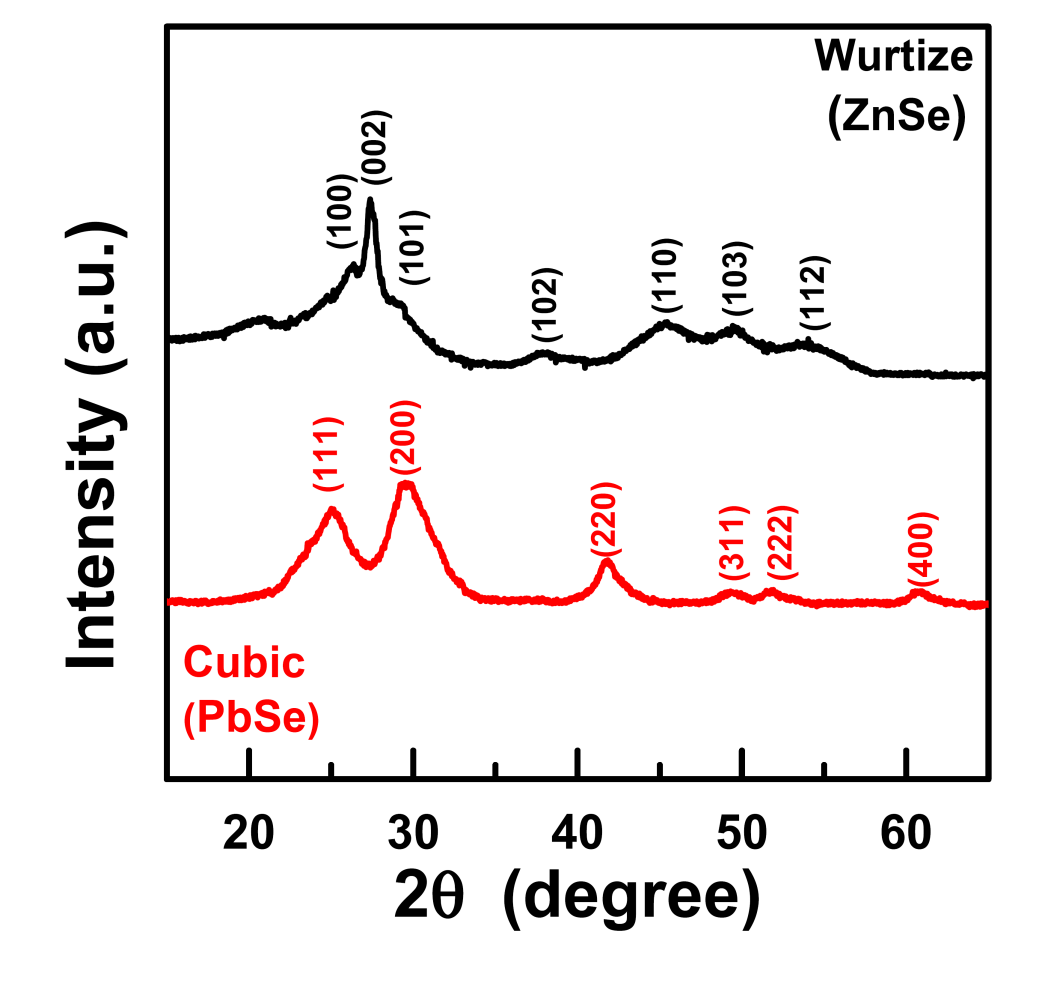


**Figure S3.** XRD patterns of ZnSe NWs and PbSe NWs.

**Figure S4.** Full-range XPS spectrum (a), XPS spectrum of Pb_4f_ core level (b), Se_3d_ core level (c) and EDS pattern (d) of resulting PbSe NWs. The peak at 142.4 eV, 137.5 eV and 54.1 eV correspond to the binding energies of Pb4f 5/2, Pb4f 7/2 and Se3d core level, respectively. Quantitative calculation of (c) and (d) gives the atomic ratio of Pb to Se in the NWs is close to 1:1, which is in good agreement with the stoichiometric ratio in PbSe.^[2]^

**Table S1.** Molar ratio and stoichiometric ratio determined by ICP analysis of ZnSe NWs and PbSe NWs.

| **Molar ratio**  **(Zn: Se)** | **Determined by ICP**  **(Zn: Se)** | **Molar ratio**  **(Pb: Se)** | **Determined by ICP**  **(Pb: Se)** |
| --- | --- | --- | --- |
| 1：1 | 0.95:1 | 1:1 | 0.93:1.0 |


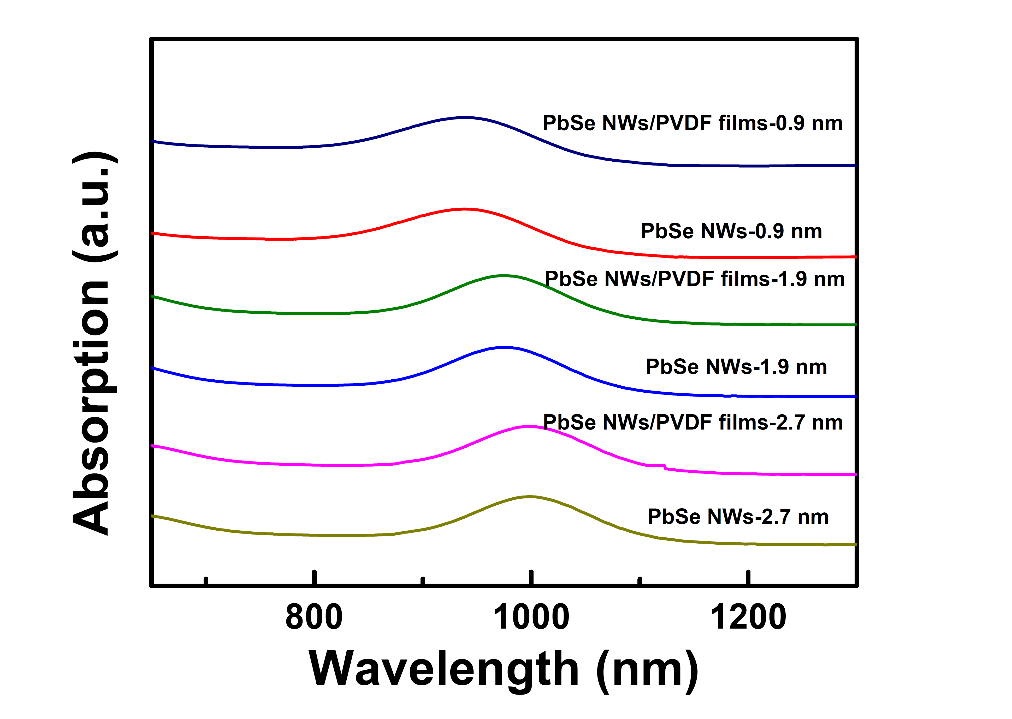


**Figure S5.** Absorption spectra of the PbSe NWs with different diameters dispersed in DMF and these PbSe NWs embedded PVDF composite films.


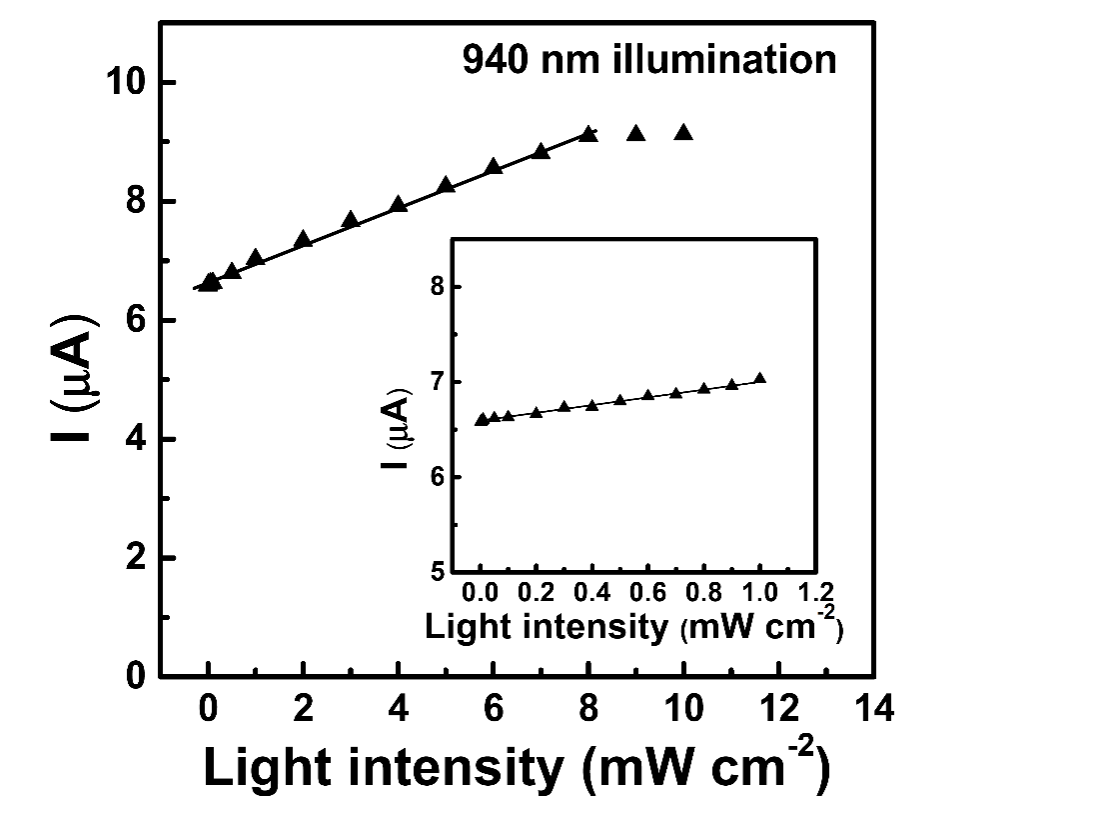


**Figure S6**. Short-circuit photocurrent density of the PEC PDs based on sub-1-nm PbSe NWs as a function of incident light intensity under 940 nm illumination. The inset is the corresponding short-circuit photocurrent density as a function of incident light intensity from 2 µW cm^-2^ to 1 mW cm^-2^.


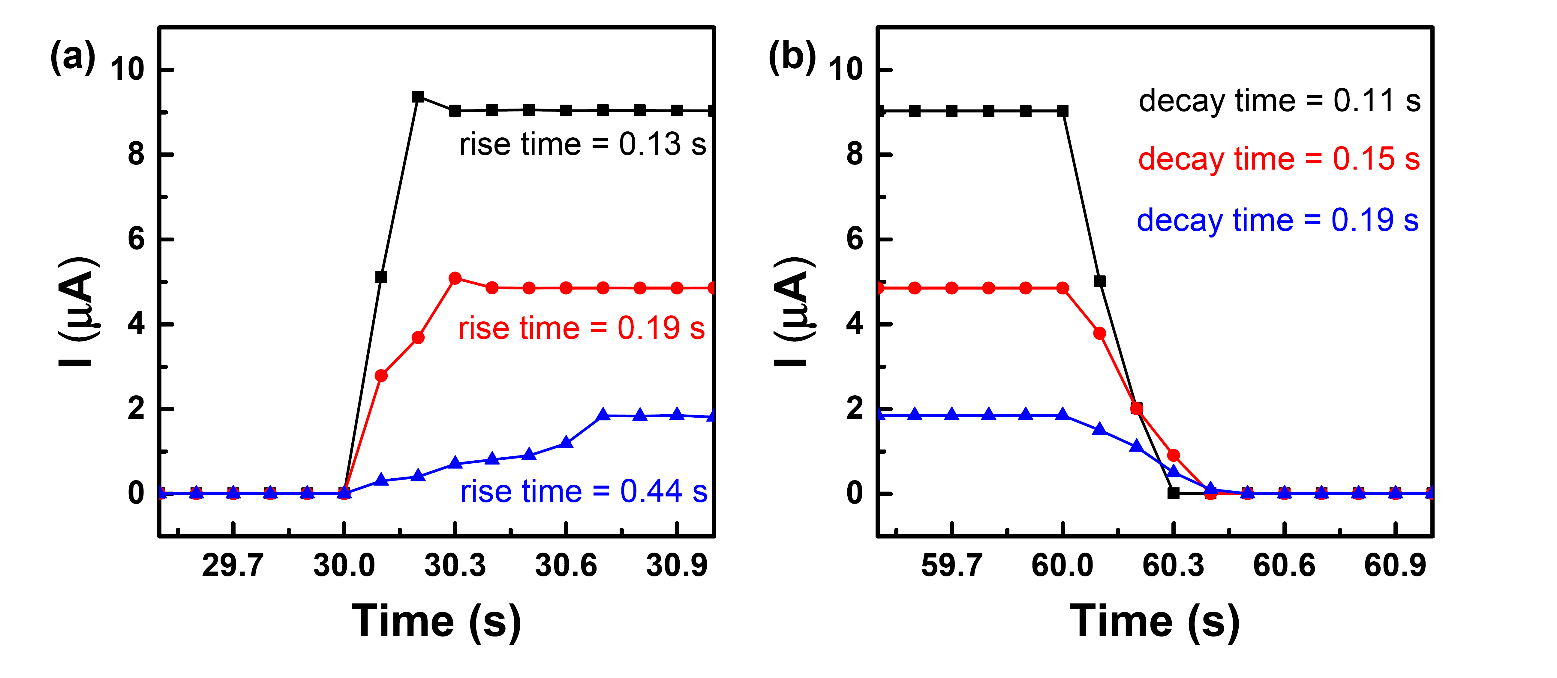


**Figure S7.** The rising edge (a) and the recovering edge (b) of the current response of the PDs based on PbSe NWs with diameters of 0.9 nm (black color), 1.9 nm (red color) and 2.7 nm (blue color). The rise time is defined as the time to reach 63% (1-1/e) of the maximum photocurrent; in contrast, decay time is defined as the time to recovery to 37% (1/e) of the maximum photocurrent. ^[3,4]^

**Figure S8.** The magnified view of the photocurrent and dark current plateau of the PDs based on sub-1-nm PbSe NWs.

**References**

[1] [D. Li](https://pubs.rsc.org/en/results?searchtext=Author:Dong%20Li),  [G. Xing](https://pubs.rsc.org/en/results?searchtext=Author:Guanjie%20Xing),  [S. Tang](https://pubs.rsc.org/en/results?searchtext=Author:Shilin%20Tang),  [X. Li](https://pubs.rsc.org/en/results?searchtext=Author:Xiaohong%20Li),  [L. Fan](https://pubs.rsc.org/en/results?searchtext=Author:Louzhen%20Fan),  [Y. Li](https://pubs.rsc.org/en/results?searchtext=Author:Yunchao%20Li), *Nanoscale* **2017**, *9*, 15044-15055.

[2] J. L. Song, W. R. Feng, Y. Ren, D. Zheng, H. Dong, R. Zhu, L. Yi, J. Hu, *Vacuum* **2018**, *155*, 1-6.

[3] J. Y. Zhou, L. L. Chen, Y. Q. Wang, Y. M. He, X. J. Pan, E. Q. Xie, *Nanoscale* **2016**, *8*, 50-73.

[4]X. D. Li, C. T. Gao, H. G. Duan, B. G. Lu, X. J. Pan, E. Q. Xie, *Nano Energy* **2012**, *1*, 640–645.
